# Supplementary material for: Measurement of the Berry curvature of solids using high-harmonic spectroscopy
Source: Nat Commun. 2018 Mar 2;9:916. doi: 10.1038/s41467-018-03397-4 (PMC5834542; doi:10.1038/s41467-018-03397-4)
Supplement: Supplementary file 1 — Supplementary Information [file 41467_2018_3397_MOESM1_ESM.pdf]

Supplementary Information for Measurement of the Berry curvature of solids using high-harmonic spectroscopy

Tran Trung Luu* and Hans Jakob Wörner
Laboratorium für Physikalische Chemie, ETH Zürich, 8093 Zürich, Switzerland
(Dated: February 7, 2018)

SUPPLEMENTARY NOTE 1: THE ROLE OF BROKEN SYMMETRY IN NONLINEAR RESPONSES

Perturbative response - expansion to different broken inversion symmetries

Although these derivations have been given before, it is convenient to present them here in a consistent formalism to provide the reader with a coherent overview of the topic (and prepare the later extension of semiclassical transport theory in solids).

Non-inversion symmetric media

Assuming that the electric field has the general form $\mathrm{E}(t) = \mathrm{E}_0\mathrm{H}(t)\sin(\omega_\mathrm{L}t)$ where E_0, $\mathrm{H}(t)$, ω_L are the peak, envelope, angular frequency of the laser electric field, the perturbative response derived from perturbation theory [1] is $\mathrm{P}_\mathrm{medium}(t) = \epsilon_0 \sum_{n=1}^{\infty} \chi^{(n)}\mathrm{E}^n(t)$ where n runs over all odd and even positive integers because of non-vanishing even-order susceptibilities in non-isotropic media [1]. In the long pulse regime, this formula can be expanded using De Moivre's formula and binomial theorem as follows where $F[X]$ is the Fourier transform of X:

$$\mathrm{P}_\mathrm{medium}(t) \propto \sum_{n=1}^{\infty} \chi^{(n)}\mathrm{E}_0^n \sin^n(\omega_\mathrm{L}t), \tag{1}$$

$$\Rightarrow F\big[\mathrm{P}_\mathrm{medium}(t)\big] \propto \sum_{m=1}^{\infty} \chi^{(m)}\mathrm{E}_0^m \Bigg(F\left[\frac{2}{2^{2m+1}}\sum_{k=0}^{m}(-1)^{(m-k)}\binom{2m+1}{k}\sin\big((2m+1-2k)\omega_\mathrm{L}t\big)\right] + F\left[\frac{1}{2^{2m}}\binom{2m}{m} + \frac{2}{2^{2m}}\sum_{k=0}^{m-1}(-1)^{(m-k)}\binom{2m}{k}\cos\big((2m-2k)\omega_\mathrm{L}t\big)\right]\Bigg), \tag{2}$$

$$\Leftrightarrow \quad \mathrm{P}_\mathrm{medium}(\omega) \propto \sum_{m=1}^{\infty} \chi^{(m)}\mathrm{E}_0^m \Bigg(\frac{2}{2^{2m+1}}\sum_{k=0}^{m}(-1)^{(m-k)}\binom{2m+1}{k}\sqrt{2\pi}\cdot\frac{\delta(\omega-a)-\delta(\omega+a)}{2i} + \frac{1}{2^{2m}}\binom{2m}{m} + \frac{2}{2^{2m}}\sum_{k=0}^{m-1}(-1)^{(m-k)}\binom{2m}{k}\sqrt{2\pi}\cdot\frac{\delta(\omega-b)+\delta(\omega+b)}{2}\Bigg),$$
$$\text{with} \quad a = (2m+1-2k)\omega_\mathrm{L},\ b = (2m-2k)\omega_\mathrm{L}. \tag{3}$$

Clearly, the Fourier transform of $\mathrm{P}_\mathrm{medium}(t)$ contains both odd and even harmonics of the fundamental carrier frequency ω_L. If the trigonometric function on the right-hand side of Supplementary Eq. 1 is of cosine type (the same electric field, with only a change of carrier-envelope phase of $\pi/2$), the expansion can be performed similarly and the result stills includes both even and odd harmonics.

Inversion-symmetric media with the electric field as the source of symmetry breaking

In this case, certainly the even-order susceptibilities vanish completely, only the odd orders $\chi^{(2n+1)}$ remain. However, the addition of the second harmonic to the fundamental electric field leads to a non-inversion-symmetric electric field: $\mathrm{E}(t) = \big(\mathrm{E}_0\sin(\omega_\mathrm{L}t) + \mathrm{E}_1\sin(2\omega_\mathrm{L}t)\big)$. Similarly, we can also expand the perturbative response of this electric field as follows:

$$\mathrm{P}_\mathrm{Et}(t) \propto \sum_{n=0}^{\infty} \chi^{(2n+1)}\big(\mathrm{E}_0\sin(\omega_\mathrm{L}t) + \mathrm{E}_1\sin(2\omega_\mathrm{L}t)\big)^{2n+1}, \tag{4}$$

$$\mathrm{P}_\mathrm{Et}(t) \propto \sum_{n=0}^{\infty} \chi^{(2n+1)}\big(\underbrace{\mathrm{E}_0^{2n+1}\sin^{2n+1}(\omega_\mathrm{L}t)}_{A} + \underbrace{\mathrm{E}_1^{2n+1}\sin^{2n+1}(2\omega_\mathrm{L}t)}_{B} + \textit{other mixing terms}\big), \tag{5}$$

The A term in Supplementary Eq. 5 is very similar to the right-hand side of Supplementary Eq. 3 except for the running index which is always odd. Therefore, it is straightforward to see that the A term includes all odd harmonics

in the spectral response. Similarly, the B term includes $2a$ and $2b$ thus all the even harmonics are included in this term. As a result, $P_{\mathrm{Et}}(\omega)$ consists of both even and odd harmonics.

The above consideration shows that regardless of the exact cause of broken symmetry (medium or electric field), as soon as the inversion symmetry in the process is broken, the polarization response will include both even and odd harmonics, although with different spectral intensities.

Origin of even and odd harmonics in the semiclassical model without anomalous term for solids

We start our discussion with the simplest model describing HHG from solids: high-energy photons are emitted due to Bragg scattering of a time-dependent electron wavepacket in a conduction band (beyond nearest-neighbor approximation) of solids. Here we follow previous works [2–7] and slightly extend recent work [8] to show how even and odd harmonics could be emitted in the framework of this semiclassical model and its implications on the quantum-mechanical model. The incident electric field is a linearly polarized 30 fs pulse at the carrier wavelength of 800 nm.

From the definition of the group velocity and the "acceleration theorem" [9, 10], which is very similar to the main text, without the anomalous terms:

$$\mathbf{v}_\nu(\mathbf{k}) = \frac{d\mathbf{r}}{dt} = \frac{1}{\hbar}\frac{\mathcal{E}_\nu(\mathbf{k})}{d\mathbf{k}}, \tag{6}$$

$$\frac{d\mathbf{k}}{dt} = -\frac{e}{\hbar}\Big(\mathbf{E} + \frac{1}{c}\mathbf{v}\times\mathbf{B}\Big), \tag{7}$$

where $\mathbf{v}_\nu(\mathbf{k}), \mathcal{E}_\nu(\mathbf{k})$ are group velocity and band dispersion of a given band ν, $\mathbf{E}$ and $\mathbf{B}$ are electric, magnetic fields. In the non-relativistic regime, $\mathbf{v}\times\mathbf{B} \ll \mathbf{E}$ thus we arrive at

$$\frac{d\mathbf{k}(t)}{dt} = -\frac{e}{\hbar}\mathbf{E}(t). \tag{8}$$

The solution of this differential equation is

$$\mathbf{k}(t) = \mathbf{k}(0) + \frac{e}{\hbar}\mathbf{A}(t), \tag{9}$$

with the vector potential $\mathbf{A}(t) = -\int_{-\infty}^{t}\mathbf{E}(t')\mathrm{d}t'$. Here we emphasize that we do not take for granted that $\mathbf{k}(0) = 0$ and we keep $\mathbf{k}(0)$ in the formula since it plays an important role in the harmonics, as will be revealed later. Since we can always decompose the band dispersion into Fourier series as

$$\mathcal{E}_\nu(\mathbf{k}) = \sum_{n=0}^{n_{\max}} \epsilon_{\nu,n}\cos(n\mathbf{k}a), \tag{10}$$

where $n_{\max}$ is the maximum number of distant neighbors considered in our model and a is the lattice constant. We should emphasize that the trigonometric function on the right-hand side of Supplementary Eq. 10 can only be of cosine type, not sine type, due to time-reversal symmetry [11], which is very important for later expansions. Inserting Supplementary Eq. 10 into Supplementary Eq. 7 we have

$$\mathbf{v}_\nu(\mathbf{k}) = -\frac{1}{\hbar}\sum_{n=0}^{n_{\max}} na\epsilon_{\nu,n}\sin(n\mathbf{k}a)., \tag{11}$$

Taking Supplementary Eq. 9 into account, we arrive at

$$\mathbf{v}_\nu(t) = -\frac{1}{\hbar}\sum_{n=0}^{n_{\max}} na\epsilon_{\nu,n}\sin\Big(na\big[\mathbf{k}(0) + \frac{e}{\hbar}\mathbf{A}(t)\big]\Big), \tag{12}$$

which can be rewritten using the trigonometric identities

$$\mathbf{v}_\nu(t) = -\frac{1}{\hbar}\sum_{n=0}^{n_{\max}} na\epsilon_{\nu,n}\Big(\sin\big[na\mathbf{k}(0)\big]\cos\big[na\frac{e}{\hbar}\mathbf{A}(t)\big] + \cos\big[na\mathbf{k}(0)\big]\sin\big[na\frac{e}{\hbar}\mathbf{A}(t)\big]\Big). \tag{13}$$

If we assume $\mathbf{A}(t) = \frac{\mathrm{E}_0}{\omega_\mathrm{L}}G(t)\cos(\omega_\mathrm{L}t + \varphi_\mathrm{CE})$ where $\frac{\mathrm{E}_0}{\omega_\mathrm{L}}$, G($t$), ω_L, φ_CE are the peak field strength, envelope, carrier angular frequency and carrier-envelope phase of the vector potential of the incident electric field, Supplementary Eq. 13 can be expanded as

$$\mathbf{v}_\nu(t) = -\frac{1}{\hbar}\sum_{n=0}^{n_{\max}} na\epsilon_{\nu,n}\Big(\sin\big[na\mathbf{k}(0)\big]\cos\big[na\frac{e}{\hbar}\frac{\mathrm{E}_0}{\omega_\mathrm{L}}\mathbf{G}(t)\cos(\omega_\mathrm{L}t+\varphi_\mathrm{CE})\big] + \cos\big[na\mathbf{k}(0)\big]\sin\big[na\frac{e}{\hbar}\frac{\mathrm{E}_0}{\omega_\mathrm{L}}\mathbf{G}(t)\cos(\omega_\mathrm{L}t+\varphi_\mathrm{CE})\big]\Big). \tag{14}$$

In the multi-cycle regime or monochromatic electric field, $\mathbf{G}(t) = 1$, we can apply the Jacobi-Anger expansion to arrive at

$$\begin{aligned}\mathbf{v}_\nu(t) = -\frac{1}{\hbar}\sum_{n=0}^{n_{\max}} na\epsilon_{\nu,n}\Bigg[&\overbrace{\sin\big[na\mathbf{k}(0)\big]\Big(J_0(na\frac{e}{\hbar}\frac{\mathrm{E}_0}{\omega_\mathrm{L}}) + 2\sum_{m=1}^{\infty}(-1)^m J_{2m}(na\frac{e}{\hbar}\frac{\mathrm{E}_0}{\omega_\mathrm{L}})\underbrace{\cos(2m\big[\omega_\mathrm{L}t+\varphi_\mathrm{CE}\big])}_{A}\Big)}^{\text{first term}}\\ &\underbrace{+\cos\big[na\mathbf{k}(0)\big]\Big(-2\sum_{m=1}^{\infty}(-1)^m J_{2m-1}(na\frac{e}{\hbar}\frac{\mathrm{E}_0}{\omega_\mathrm{L}})\overbrace{\cos\big[(2m-1)\big[\omega_\mathrm{L}t+\varphi_\mathrm{CE}\big]\big]}^{B}\Big)}_{\text{second term}}\Bigg].\end{aligned} \tag{15}$$

Here J_l is the Bessel function of the first kind of order l. Now the associated spectrum of the intraband current can be calculated as proportional to $\big|F[\mathbf{v}_\nu(t)]\big|^2$. This can be calculated through $F[A]$ and $F[B]$ where

$$F[A] = \sqrt{2\pi}\cdot\frac{\delta(\omega-2m\omega_\mathrm{L})+\delta(\omega+2m\omega_\mathrm{L})}{2}\cos(2m\varphi_\mathrm{CE}) - \sqrt{2\pi}\cdot\frac{\delta(\omega-2m\omega_\mathrm{L})-\delta(\omega+2m\omega_\mathrm{L})}{2i}\sin(2m\varphi_\mathrm{CE}), \tag{16}$$

$$F[B] = \sqrt{2\pi}\cdot\frac{\delta(\omega-(2m-1)\omega_\mathrm{L})+\delta(\omega+(2m-1)\omega_\mathrm{L})}{2}\cos((2m-1)\varphi_\mathrm{CE}) - \sqrt{2\pi}\cdot\frac{\delta(\omega-(2m-1)\omega_\mathrm{L})-\delta(\omega+(2m-1)\omega_\mathrm{L})}{2i}\sin((2m-1)\varphi_\mathrm{CE}). \tag{17}$$

Equations 15, 16, 17 comprise the full analytical solution of the semiclassical model under minimal approximation. Two conclusions can be drawn at this stage:

- The spectrum associated with the intraband current consists of modulated Dirac combs with both even and odd harmonics (represented in Supplementary Eq. 16 and 17) which are included in the first term and second term respectively, under general conditions.
- The spectral intensity of the harmonics depends on $J_l, n, \epsilon_{\nu,n}, a, \frac{\mathrm{E}_0}{\omega_\mathrm{L}}$, but are independent of φ_CE because $|F[A]|^2_{\omega=l\omega_\mathrm{L}} = |F[B]|^2_{\omega=l\omega_\mathrm{L}} = \pi/2$, which is expected for a long pulse.

And the consequences of these equations are

- It is important to remember that time-reversal symmetry results in a symmetric (even function) band dispersion, thus the trigonometric function in Supplementary Eq. 12 is of sine type. If time-reversal symmetry was not in force, the trigonometric function in Supplementary Eq. 12 would be of cosine type hence the first term in Supplementary Eq. 15 would contain odd harmonics whereas the second term would contain even harmonics. The result is very similar to the case of even harmonics due to Berry curvature as discussed in [12].
- In a typical semiclassical model, it is natural to start with $\mathbf{k}(0) = 0$ thus $\sin\big[na\mathbf{k}(0)\big] = 0$, $\cos\big[na\mathbf{k}(0)\big] = 1$ hence first term in Supplementary Eq. 15 vanishes completely and the second term is maximized. This means that the even harmonics are canceled entirely, only the odd harmonics remain. Therefore, a typical semiclassical simulation in this case will result in a spectrum of purely odd harmonics.
- If for any reason, the initial electron wavepacket in the semiclassical model does not start at 0 which means $\mathbf{k}(0) \neq 0$, both the first term and the second term will exist, the emitted spectrum will inevitably contain both odd and even harmonics. This result is best illustrated in Supplementary Figure 1 using the semiclassical model [8].

Odd and even harmonics in the semiclassical model with anomalous term (Berry curvature)

Here we consider an extended version of Supplementary Eq. 6 which is Eq. 1 in the main text.

$$\mathbf{v}_\nu(\mathbf{k}) = \underbrace{\frac{1}{\hbar}\frac{\mathcal{E}_\nu(\mathbf{k})}{d\mathbf{k}}} - \underbrace{\frac{e}{\hbar}\mathbf{E}(t)\times\mathbf{\Omega}_\nu(\mathbf{k})}, \tag{18}$$

$$\Leftrightarrow \qquad v_\nu(t) = v_\nu(t)_{\parallel} \quad + \quad v_\nu(t)_{\perp}. \tag{19}$$

where $v_\nu(t)_{\parallel}$ is the ordinary, parallel component of the current that has the full expansion described in Supplementary Eq. 15. Similarly we can expand the second term $v_\nu(t)_\perp$ using the Fourier expansion of $\mathbf{\Omega}_\nu(\mathbf{k})$ (Eq. 2 in the main text) as:

$$v_\nu(t)_\perp = \frac{e}{\hbar}\overbrace{\mathrm{E}_0\mathbf{H}(t)\sin(\omega_\mathrm{L}t+\varphi_\mathrm{CE})}^{\mathbf{E}(t)}\cdot\overbrace{\sum_{n=1}^{\infty}\gamma_n\sin\Big(na\big[\mathbf{k}(0)+\frac{e}{\hbar}\mathbf{A}(t)\big]\Big)}^{\mathbf{\Omega}_\nu(\mathbf{k})}, \tag{20}$$

$$\Leftrightarrow v_\nu(t)_\perp = \frac{e\mathrm{E}_0}{\hbar}\sum_{n=1}^{\infty}\gamma_n\mathbf{H}(t)\sin(\omega_\mathrm{L}t+\varphi_\mathrm{CE})\Big(\sin\big[na\mathbf{k}(0)\big]\cos\big[na\frac{e}{\hbar}\mathbf{A}(t)\big]+\cos\big[na\mathbf{k}(0)\big]\sin\big[na\frac{e}{\hbar}\mathbf{A}(t)\big]\Big). \tag{21}$$

Now for simplicity, we assume that $\mathrm{H}(t) = \mathrm{G}(t) = 1$ and $\varphi_\mathrm{CE} = 0$, Supplementary Eq. 21 reduces to:

$$v_\nu(t)_\perp = \frac{e\mathrm{E}_0}{\hbar}\sum_{n=1}^{\infty}\gamma_n\sin(\omega_\mathrm{L}t)\Big(\sin\big[na\mathbf{k}(0)\big]\cos\big[na\frac{e}{\hbar}\frac{\mathrm{E}_0}{\omega_\mathrm{L}}\cos(\omega_\mathrm{L}t)\big]+\cos\big[na\mathbf{k}(0)\big]\sin\big[na\frac{e}{\hbar}\frac{\mathrm{E}_0}{\omega_\mathrm{L}}\cos(\omega_\mathrm{L}t)\big]\Big), \tag{22}$$

$$\begin{aligned} &= \frac{e\mathrm{E}_0}{\hbar}\sum_{n=1}^{\infty}\gamma_n\Bigg[\sin\big[na\mathbf{k}(0)\big]\sin(\omega_\mathrm{L}t)\Big(J_0(na\frac{e}{\hbar}\frac{\mathrm{E}_0}{\omega_\mathrm{L}})+2\sum_{m=1}^{\infty}(-1)^m J_{2m}(na\frac{e}{\hbar}\frac{\mathrm{E}_0}{\omega_\mathrm{L}})\cos(2m\omega_\mathrm{L}t)\Big) \\ &+\cos\big[na\mathbf{k}(0)\big]\sin(\omega_\mathrm{L}t)\Big(-2\sum_{m=1}^{\infty}(-1)^m J_{2m-1}(na\frac{e}{\hbar}\frac{\mathrm{E}_0}{\omega_\mathrm{L}})\cos\left[(2m-1)\,\omega_\mathrm{L}t\right]\Big)\Bigg]. \end{aligned} \tag{23}$$

We consider the limit where $\mathbf{k}(0) = 0$ then Supplementary Eq. 23 reduces to:

$$v_\nu(t)_\perp = \frac{e\mathrm{E}_0}{\hbar}\sum_{n=1}^{\infty}\gamma_n\Bigg[\sin(\omega_\mathrm{L}t)\Big(-2\sum_{m=1}^{\infty}(-1)^m J_{2m-1}(na\frac{e}{\hbar}\frac{\mathrm{E}_0}{\omega_\mathrm{L}})\cos\left[(2m-1)\,\omega_\mathrm{L}t\right]\Big)\Bigg], \tag{24}$$

$$= \frac{e\mathrm{E}_0}{\hbar}\sum_{n=1}^{\infty}\gamma_n\Bigg[-\sum_{m=1}^{\infty}(-1)^m J_{2m-1}(na\frac{e}{\hbar}\frac{\mathrm{E}_0}{\omega_\mathrm{L}})\Big(\sin\left[2m\omega_\mathrm{L}t\right]+\sin\left[(2m-2)\omega_\mathrm{L}t\right]\Big)\Bigg]. \tag{25}$$

The associated emitted spectra can be calculated using $F\big[v_\nu(t)_\perp\big]$ as:

$$\begin{aligned} v_\nu(\omega)_\perp = \frac{e\mathrm{E}_0}{\hbar}\sum_{n=1}^{\infty}\gamma_n\Bigg[&-\sum_{m=1}^{\infty}(-1)^m J_{2m-1}(n\frac{\Omega_B}{\omega_\mathrm{L}})\cdot \\ &\Big(\sqrt{2\pi}\cdot\frac{\delta(\omega-2m\omega_\mathrm{L})-\delta(\omega+2m\omega_\mathrm{L})}{2i}+\sqrt{2\pi}\cdot\frac{\delta(\omega-(2m-2)\omega_\mathrm{L})-\delta(\omega+(2m-2)\omega_\mathrm{L})}{2i}\Big)\Bigg], \end{aligned} \tag{26}$$

where $\Omega_B = ae\mathrm{E}_0/\hbar$ is the Bloch frequency. Supplementary Eq. 26 is the general expression in the long pulse limit of anomalous current due to non-vanishing Berry curvature, which is very similar to [12]. Clearly, the associated spectrum contains only even harmonics of the fundamental laser frequency. The most important message from this derivation is that the broken symmetry of the medium results in an odd function of the Berry curvature, therefore its trigonometric function is of sine type. This together with the Jacobi-Anger expansion leads to Supplementary Eq. 25,26 which contain only even harmonics.

Semiclassical simulations

As a simple illustration for the consideration of the broken symmetry in the semiclassical model, we performed numerical simulations [8, 13] for a linearly polarized 30 fs pulse at the carrier wavelength of 800 nm, peak electric field strength 0.5 V/Å and the results are shown in Supplementary Figure 1.

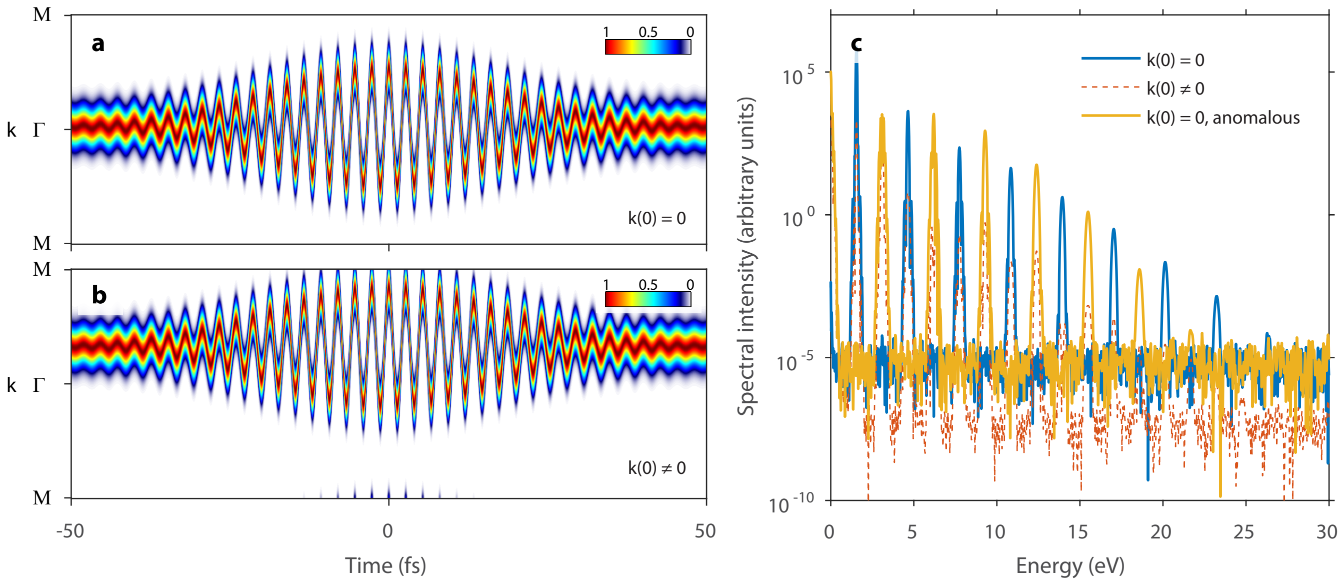

Supplementary Figure 1. **Odd and even harmonics in the semiclassical model for an incident linearly polarized electric field**. **a,b,** Temporal evolution of the electron wavepacket probably density in the conduction band, with $\mathbf{k}(0) = 0$ and $\mathbf{k}(0) \neq 0$. **c,** Associated emitted spectra of the two cases and $\mathbf{k}(0) = 0$ with anomalous velocity . The electric field used is a 30 fs pulse at 800 nm.

In a typical case where $\mathbf{k}(0) = 0$, the associated spectrum of Supplementary Figure 1a consists of purely odd harmonics (Supplementary Figure 1c, solid dark blue line). On the other hand, if $\mathbf{k}(0) \neq 0$ then the associated spectrum consists of both odd and even harmonics, Supplementary Figure 1c dashed red line. If $\mathbf{k}(0) = 0$ and Berry curvature is not zero, then the associated spectrum consists of only even harmonics, Supplementary Figure 1c solid orange line. In this particular simulation the Berry curvature is simulated as: $\Omega(k) = \sin(ka) + \sin(2ka) + \sin(3ka) + \sin(4ka)$.

SUPPLEMENTARY NOTE 2: EXTREME–ULTRAVIOLET POLARIZER

Design

There are several requirements for constructing the EUV polarizer which would be suitable for our experiments:

- The polarizer should be able to transmit a broad photon energy range, preferably much broader than the range of our experiments: $0 - 30$ eV, well in the vacuum ultraviolet regime.
- The polarizer should have a high contrast between the horizontal and vertical polarization.
- The polarizer should not change the direction of the output beam as compared to the input beam so that the energy calibration of the whole spectrometer during polarizer's measurements would not be an issue.

The first requirement results in the use of metallic mirrors at grazing incidence. The grazing-incidence angle is optimized such that the total throughput and contrast are maximized (second requirement). In order to satisfy the third requirement, a symmetric assembly should be used. The final design consists of four bare gold mirrors, all at grazing angle of 20 degrees, assembled as illustrated in Supplementary Figure 2a and main text, Fig. 2a. The whole assembly is placed on a rotary stage that has the rotation axis identical to the propagation axis of the laser beam, which satisfies the third requirement.

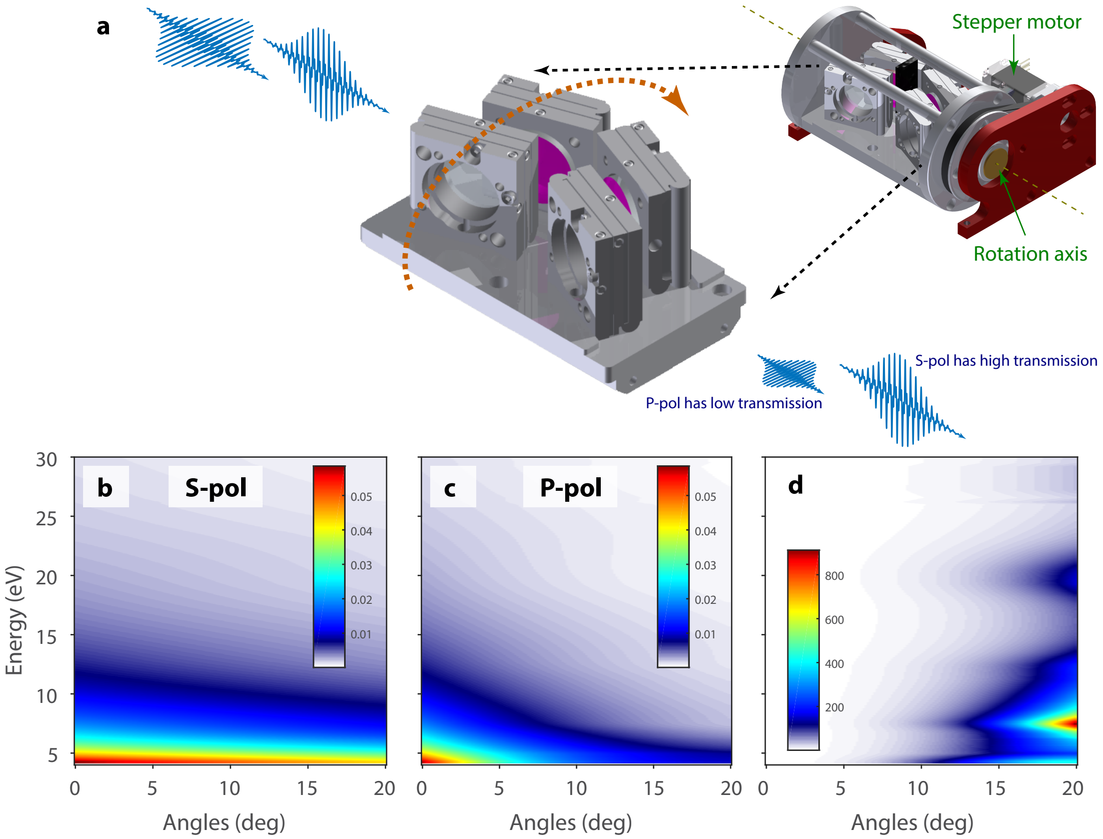

Supplementary Figure 2. **All reflective extreme–ultraviolet polarizer design**. **a,** Three dimensional drawing of the rotary EUV polarizer. The whole assembly is put on a rotary stage with the rotation axis overlapping with the incident electric field k-vector. The linearly polarized input pulse will have a much higher throughput if it is S-pol, compared to P-pol. **b,c,** Calculated reflectivity of the EUV polarizer for different input polarizations after one reflection. **d,** Enhancement ratio between S-pol and P-pol after four reflections.

In this configuration, the linearly polarized input electric field after one reflection on S-pol (Supplementary Figure 2b) is stronger than the one on P-pol (Supplementary Figure 2c), illustrated in Supplementary Figure 2a. The reflectivity data of bare gold is taken from standard source [14]. The enhancement factor (or contrast) is calculated as the total reflectivity for S-pol divided by the total reflectivity for P-pol and plotted in Supplementary Figure 2d after four reflections. Here, the calculated contrast between the S-pol and P-pol beam at 17 eV (11th harmonic) is ≈ 70 times.

Calibration

In order to properly examine the polarization of the HHG spectra from solids, the polarizer contrast should be calibrated. Its calibration is done by performing measurements of HHG from gases under identical experimental conditions. It is known that HHG from interaction of noble gases with linearly polarized multi-cycle pulses is linearly polarized, parallel to the polarization of the input pulse. We performed polarization measurement of HHG from xenon under high contrast linearly polarized input pulses and the result is shown in Supplementary Figure 3a.

The cross-cut of the spectrogram at 11th harmonics (17 eV) shows the contrast, spectral intensity at parallel polarization divided by spectral intensity measured in perpendicular polarization, of ≈ 60. Because the polarization purity of the incident laser electric field is very high, more than three orders of magnitude, we can conclude that the contrast of our polarizer at 17 eV is $60 : 1$.

Since the experiments in the main text show the contrast of $30 : 1$ at 17 eV, it means that the remaining perpendicular polarization accounts for not more than $2/60$ which is $\approx 3.3\%$ of the total spectral intensity. Therefore all polarizations measured in the main text are linearly polarized, with a depolarization ratio of less than 4%.

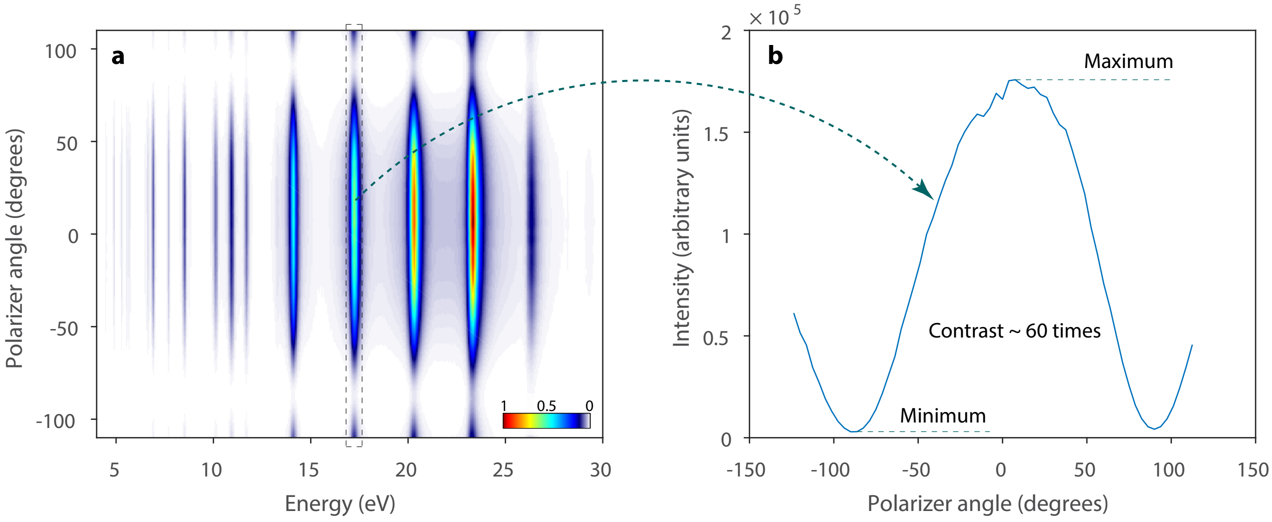

Supplementary Figure 3. **Contrast calibration of the EUV polarizer**. **a,** Recorded spectra as a function of the polarizer angle, emitted from xenon, pumped by 30 fs pulses at the carrier wavelength of 800 nm. **b,** Cross-cut of the 11th harmonic showing the maximum contrast of ≈ 60 times.

SUPPLEMENTARY NOTE 3: AB–INITIO CALCULATIONS OF THE BERRY CURVATURE IN SIO_2 - α–QUARTZ

Methodologies

Although the ideas related to Berry phase and curvature have been developed over the last three decades, actual calculations of Berry phase and curvature of condensed-matter systems only recently gained interest due to development in studies of electronic charge and spin transport of electrons. Here we outline three methodologies for calculating the Berry curvature that are used in our work. Interested readers could consult [15] for a more comprehensive review on this topic.

Direct implementation

In solid-state physics, the parameter space $\mathbf{R}$ on which the quantum system evolves is $\mathbf{k}$-space. Thus the generic Berry curvature $\mathbf{\Omega}_n(\mathbf{R})$ is represented by $\mathbf{\Omega}_n(\mathbf{k})$ where n is a quantum number specifying the band index and $\mathbf{k}$ is the crystal momentum. The definitions of the Berry connection $\mathbf{A}_n(\mathbf{k})$ and Berry curvature $\mathbf{\Omega}_n(\mathbf{k})$ [15–17] are given by

$$\mathbf{A}_n(\mathbf{k}) = i\int_{uc} \mathrm{d}^3 r u^*_{n,\mathbf{k}}(\mathbf{r})\nabla_{\mathbf{k}} u_{n,\mathbf{k}}(\mathbf{r}), \tag{27}$$

$$\mathbf{\Omega}_n(\mathbf{k}) = \nabla_{\mathbf{k}} \times \mathbf{A}_n(\mathbf{k}),$$

$$\mathbf{\Omega}_n(\mathbf{k}) = i\int_{uc} \mathrm{d}^3 r \nabla_{\mathbf{k}} u^*_{n,\mathbf{k}}(\mathbf{r}) \times \nabla_{\mathbf{k}} u_{n,\mathbf{k}}(\mathbf{r}), \tag{28}$$

where $u_{n,\mathbf{k}}(\mathbf{r})$ is the cell-periodic part of the Bloch wavefunction $\Psi_{n,\mathbf{k}}(\mathbf{r})$. The integral in Supplementary Eq. 28 is performed over the whole unit cell, spanned by the $(\hat{\mathbf{a}}_1, \hat{\mathbf{a}}_2, \hat{\mathbf{a}}_3)$ primitive vectors. Since the derivative operation is done in $\mathbf{k}$-space, the direct implementation suffers from the fact that a large number of ab–initio calculations are needed in order to converge the calculation of Supplementary Eq. 28, which in extreme cases can reach up to one million $\mathbf{k}$-points [15].

Kubo formula

In order to overcome the expensive calculation cost induced by the direct implementation, there have been multiple methodologies proposed [15, 17]. One widely used methodology is the use of a Kubo-like formula [18, 19]:

$$\mathbf{\Omega}_n(\mathbf{k}) = i\hbar^2 \sum_{m\neq n} \frac{\langle \Psi_{n,\mathbf{k}}|\, \hat{\mathbf{v}}\, |\Psi_{m,\mathbf{k}}\rangle \times \langle \Psi_{m,\mathbf{k}}|\, \hat{\mathbf{v}}\, |\Psi_{n,\mathbf{k}}\rangle}{(\varepsilon_{n,\mathbf{k}} - \varepsilon_{m,\mathbf{k}})^2}. \tag{29}$$

Here, $\hat{\mathbf{v}}$ is the usual velocity operator, $\varepsilon_{i,\mathbf{k}}$ is the eigen-energy of the band index i and crystal momentum $\mathbf{k}$, and for compact representation, the Dirac notation is used. Equation 29 replaces the computationally demanding derivative operation in Supplementary Eq. 28 by evaluation of the velocity matrix element and the loop through all the band indices $\{i\}$. Theoretically, all the band indices (occupided and unoccupied states) have to be included in the evaluation of Supplementary Eq. 29. However, practically only a small number of bands are needed as we will show in the next section.

Fukui formula

Another good method that we employed in our calculation is a technique introduced by Fukui, Hatsugai, and Suzuki [20, 21] and being used later [22, 23] by defining a so-called link variable:

$$U_{\mu,n}(\mathbf{k}) = \frac{\langle u_{n,\mathbf{k}} | u_{n,\mathbf{k}+\hat{\mu}}\rangle}{|\langle u_{n,\mathbf{k}} | u_{n,\mathbf{k}+\hat{\mu}}\rangle|}, \tag{30}$$

where $\hat{\mu}$ is the vector in the direction $\mu = (\hat{\mathbf{k}}_x, \hat{\mathbf{k}}_y, \hat{\mathbf{k}}_z)$ with the fractional amplitude $1/(N_\mu - 1)$ with N_μ being the number of $\mathbf{k}$-points used in discretizing the Brillouin zone in μ direction. Next, the lattice field strength is defined by

$$F_{12,n}(\mathbf{k}) = \ln \frac{U_{1,n}(\mathbf{k}) U_{2,n}(\mathbf{k} + \hat{\mathbf{1}})}{U_{1,n}(\mathbf{k} + \hat{\mathbf{2}}) U_{2,n}(\mathbf{k})}, \tag{31}$$

and for large N_μ, the corresponding Berry curvature will be calculated as

$$\Omega_{3,n}(\mathbf{k}) = \frac{\operatorname{Im} F_{12,n}(\mathbf{k})}{A_{\text{plaquette}}}, \tag{32}$$

with the area of the plaquette $A_{\text{plaquette}} = \frac{\hat{\mathbf{k}}_x \cdot \hat{\mathbf{k}}_y}{(N_1-1)(N_2-1)}$ where $(\hat{\mathbf{k}}_x, \hat{\mathbf{k}}_y, \hat{\mathbf{k}}_z)$ are primitive vectors in the reciprocal lattice. This method has been proven [20, 21] to be extremely stable even for a very small number of $\mathbf{k}$-points.

Numerical considerations

Since SiO_2 ($\alpha-$quartz) has a hexagonal lattice, not the simple cubic lattice with orthogonal primitive vectors, thus $(\hat{\mathbf{k}}_x, \hat{\mathbf{k}}_y) = 60^\circ$ and $(\hat{\mathbf{k}}_x, \hat{\mathbf{k}}_z) = (\hat{\mathbf{k}}_y, \hat{\mathbf{k}}_z) = 90^\circ$ as shown in the main text, Fig. 2b, and vectorial $\mathbf{k}$ is used throughout the calculations. Here, the direct implementation and Kubo formula result in a full vectorial Berry curvature $\mathbf{\Omega}_n(\mathbf{k}) = (\Omega_{x,n}(\mathbf{k}), \Omega_{y,n}(\mathbf{k}), \Omega_{z,n}(\mathbf{k}))$ whereas the Fukui formula gives the result in only one predefined direction $\Omega_{i,n}(\mathbf{k})$ where $i = (x, y, z)$. Within the scope of our work, the quantity of interest is $\Omega_{z,n}(\mathbf{k})$ for all $\mathbf{k}$ points making the directions $\Gamma - \mathrm{M}$ and $\Gamma - \mathrm{K}$ with the corresponding fractional $\mathbf{k}$-points $(0,0,0) - (0,0.5,0)$ and $(0,0,0) - (1/3,1/3,0)$. Because there are 3 silicon atoms and 6 oxygen atoms in a unit cell, there are 48 valence electrons accordingly contributing to the valence bands. Due to spin, each band is doubly degenerate thus there are in total 24 valence bands. Our focus is on the bands close to the Fermi level which has the quantum number n close to 24 (top valence band) and 25 (bottom conduction band). The focus of our calculation is the first conduction band in $\Gamma - \mathrm{M}$ direction where there is no degeneracy with other bands, leaving a smooth band dispersion and accordingly no sharp jump in the Berry curvature.

For the direct implementation, we used 6th order central finite difference for the evaluation of the derivation. Combining this with three different $\mathbf{k}$ directions, it results into 18 different sets of $u_{n,\mathbf{k}}(\mathbf{r})$ and corresponding Bloch

wavefunctions $\Psi_{n,\mathbf{k}}(\mathbf{r})$ for each $\mathbf{k}$ point and a given band index. The step size for the derivative evaluation is different (much smaller) than the step size used for the evaluation of the Berry curvature on the whole $\mathbf{k}$ points spanning the direction of interest.

For the Kubo formula, there is no derivation in $\mathbf{k}$-space, but instead an evaluation of the velocity matrix elements which can be easily carried out using Fast Fourier Transform. In addition, all of the band indices need to be included thus the total number of Bloch wavefunctions required is not small.

For the Fukui formula, only one band index is needed but due to Supplementary Eq. 30, 31, 8 different sets of $u_{n,\mathbf{k}}(\mathbf{r})$ are required for evaluation of $\Omega_{i,n}$ at one $\mathbf{k}$ point.

All calculations are performed at room temperature (300K).

Convergence

For all calculations reported in this manuscript, the self-consistent calculation was performed first using an existing software package [24, 25], then the calculation of the Bloch wavefunctions is done using $\mathbf{k}$-meshes of different sizes to test for convergence. The results are presented in Supplementary Figure 4.

As it is expected from the nature of the direct implementation and the Kubo formula, there is no dependence of the Berry curvature calculation on the size of the grid that spans $\Gamma - \mathrm{M}$, Supplementary Figure 4a,c. However, if the fractional dk is 10^{-4} or larger, the calculation does not converge, as shown in Supplementary Figure 4b. Therefore, convergence of the direct implementation is reached when the fractional dk is 10^{-6} or smaller which is very compatible with the requirement mentioned in [15]. For the Kubo formula, as mentioned in the previous section, evaluation of Supplementary Eq. 29 is dependent on the number m of the bands taken into account. Figure S 4d shows that as long as more than 7 bands below and 7 bands above the desired band are taken into account, the calculation is converged. Nevertheless, for the Fukui formula, the standard deviation changes as a function of the grid size. Reliable results can only be achieved if the Brillouin zone is discretized by 100 or more $\mathbf{k}$ points. For speed and convenience reasons, the direct approach is the method of choice for any further calculations.

Dependence on exchange-correlation energy functional approximations

Supplementary Figure 5a,b,c show how different exchange-correlation approximations affect the bandstructure and consequently the calculated Berry curvature (Supplementary Figure 5d). While it was well known that the introduction of the modified Becke Johnson exchange potential with correlation effects [26] should result in a more accurate bandgap. However, the bandgaps retrieved in all approximations here are different from the experimentally measured one of about 9 eV [27, 28]. This reveals the shortcommings of the LCAO approach as compared to the full-potential (linearized) augmented plane-wave and local orbitals (FP-(L)APW + lo) method as used in [26, 29]. For this reason, our experiment and follow-up developments provide a new approach to benchmark ab–initio calculations.

Berry curvature calculated for $\Gamma - \mathrm{M}$ and $\Gamma - \mathrm{K}$

It is intuitive to note that the valence bands 1 and 2 (VB1, 2) and conduction bands 2 and 3 (CB2, 3) have degeneracies at the Γ point as shown in Supplementary Figure 6a, thus their Berry curvatures are a bit higher close to the Γ point. At the same time, they have almost opposite Berry curvature values, as shown in Supplementary Figure 6b. In addition, if we consider the fact that VB3 is more likely to be filled than CB1 to be populated, this leaves CB1 as the most effective band, influencing Berry phase effects in this direction which would correspond to the $\Omega_{\text{effective}}(\mathbf{k})$ in the main text. Furthermore, Supplementary Figure 6c shows that the Berry curvature of the same band is very much (100 times) weaker in the $\Gamma - \mathrm{K}$ direction. For symmetry reason, it should be zero. In the semiclassical model, this translates into a spectral intensity that is about four orders of magnitude less intense. This is certainly too weak to be seen within the precision of our measurements.

A direct comparison between the experimentally retrieved Berry curvature and the theoretically calculated value is shown in the main text, Fig. 3b. Near-quantitative agreement is obtained with the MGGA functional (shown) and the LDA functional (not shown), whereas the GGA functional appears to overestimate the Berry curvature by a factor of ≈ 2. Discrepancy of the values can be attributed to: (i) imperfections of the experimental measurements, (ii) first order derivation of the adiabatic transport theory utilized in determining the retrieved value from experimental results, and (iii) limitations inherent to the ab–initio calculations: even in the DFT:LCAO technique, different functionals used already lead to amplitude variations up to a factor of ≈ 2. (Supplementary Figure 5d, LDA-PZ versus MGGA-TB09LDA).

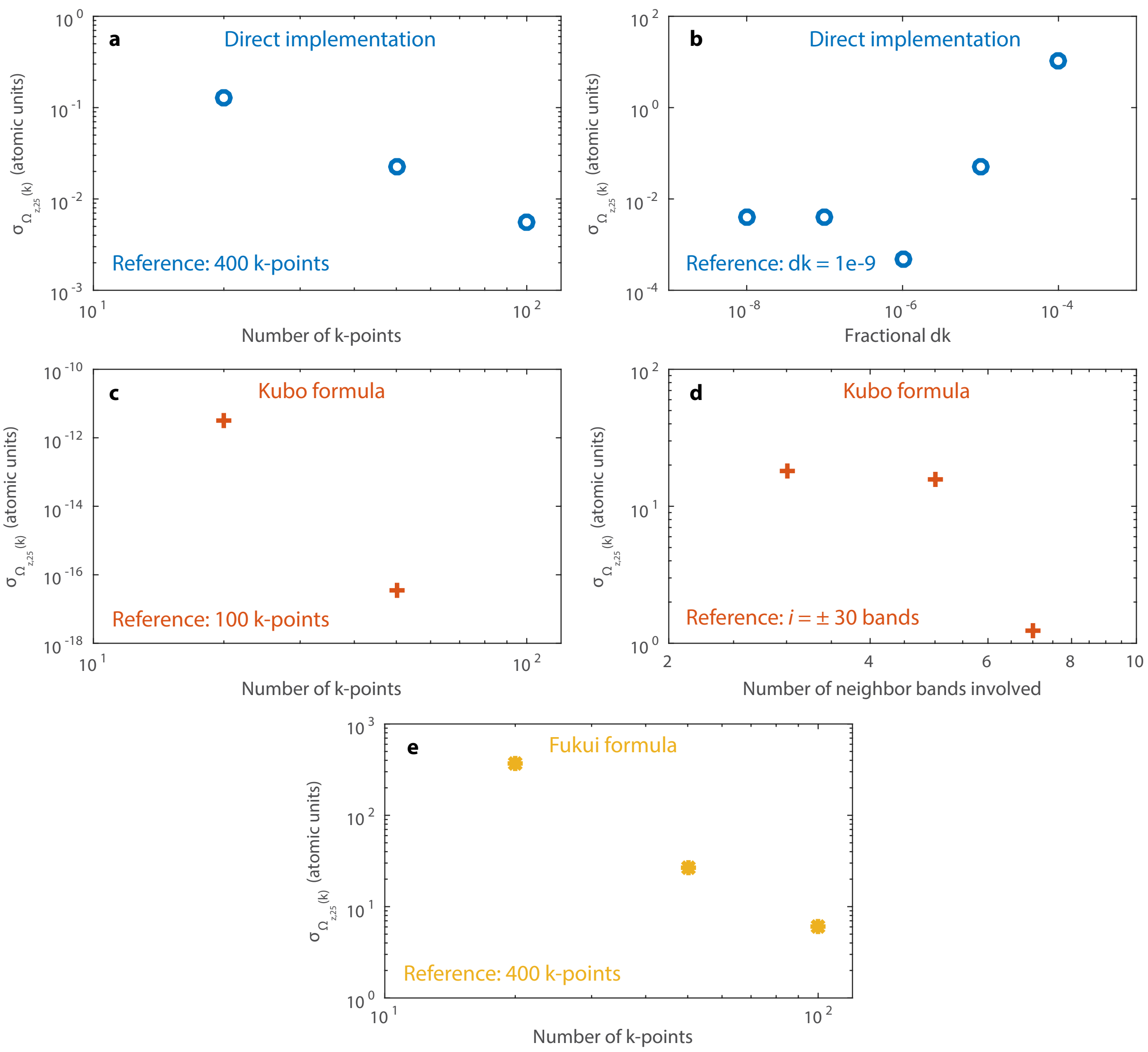

Supplementary Figure 4. **Convergence of Berry curvature calculations of the first conduction band. a, c, e**, Standard deviation of the Berry curvature as a function of the number of **k** points spanning the direction $\Gamma - \mathrm{M}$ for three methods. **b,** For direct implementation, standard deviation as a function of the fractional dk used in the derivative evaluation. **d,** For Kubo formula, standard deviation as a function of the number of neighboring bands used in Supplementary Eq. 29: i bands above and i bands below with $i = 3, 5, 7$. All the references are calculations at the highest discretization size possible.

SUPPLEMENTARY NOTE 4: DENSITY OF STATES

Supplementary Figure 7 shows the calculated density of states in SiO_2 crystal using different approximations. Because the density of states expands more than 30 eV with a relatively constant amplitude for both crystal directions, this is not in favor of the picture of multiple plateaus due to higher-lying conduction bands [30, 31]. As a result, more extensive experiments and simulations should be carried out in order to fully explain the second plateau behavior in HHG from quartz.

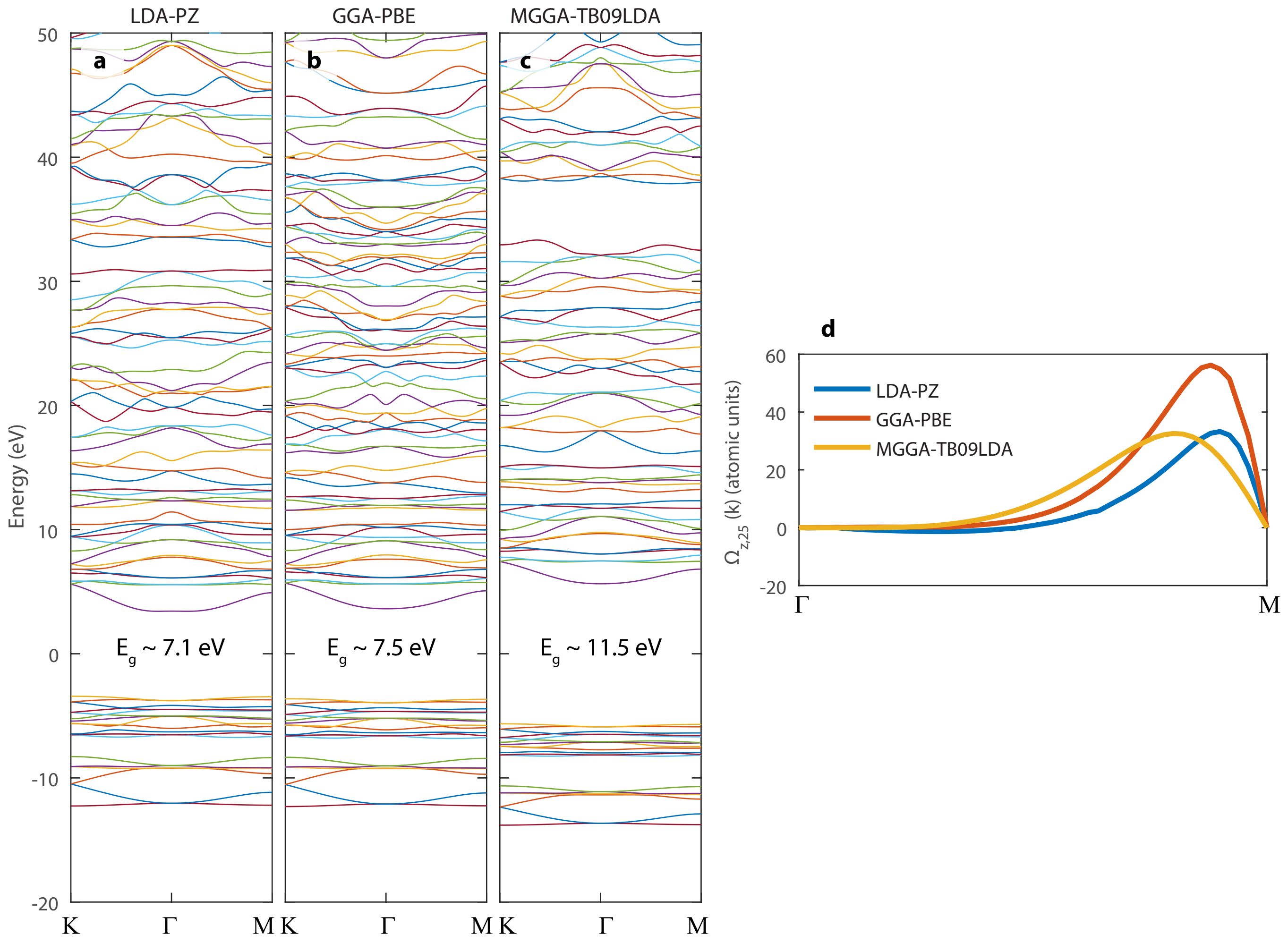

Supplementary Figure 5. **Berry curvature calculation for different exchange-correlation functionals. a, b, c**, Bandstructure calculated using different approximations (LDA, GGA, MGGA) and different parameterizations. The corresponding direct bandgaps are illustrated. **d**, Berry curvature calculated using the direct approach with three different functionals.

SUPPLEMENTARY NOTE 5: FURTHER TECHNICAL INFORMATION

Toward a precise determination of the spectral intensity

In many cases where the absolute spectral intensity is not needed, only the relative intensity is of importance, for instance in Fig. 2b,c,d of the main text, we do not need to calibrate the spectral intensity perfectly. However, for the application of retrieving the Berry curvature, accurate spectral intensity is needed. In order to determine the spectral intensity as precisely as possible, we took care of the following issues:

Wavelength calibration

Since the spectrometer is grating-based, we perform the calibration in wavelength domain first, then the spectrum is converted to frequency domain for plotting. By measuring the spectrum of the incident light, the carrier central wavelength is determined to be $\lambda_0 \approx 798.9$ nm. We recorded HHG spectra from gases and solid samples. The formed high-harmonics are calibrated using the grating equation.

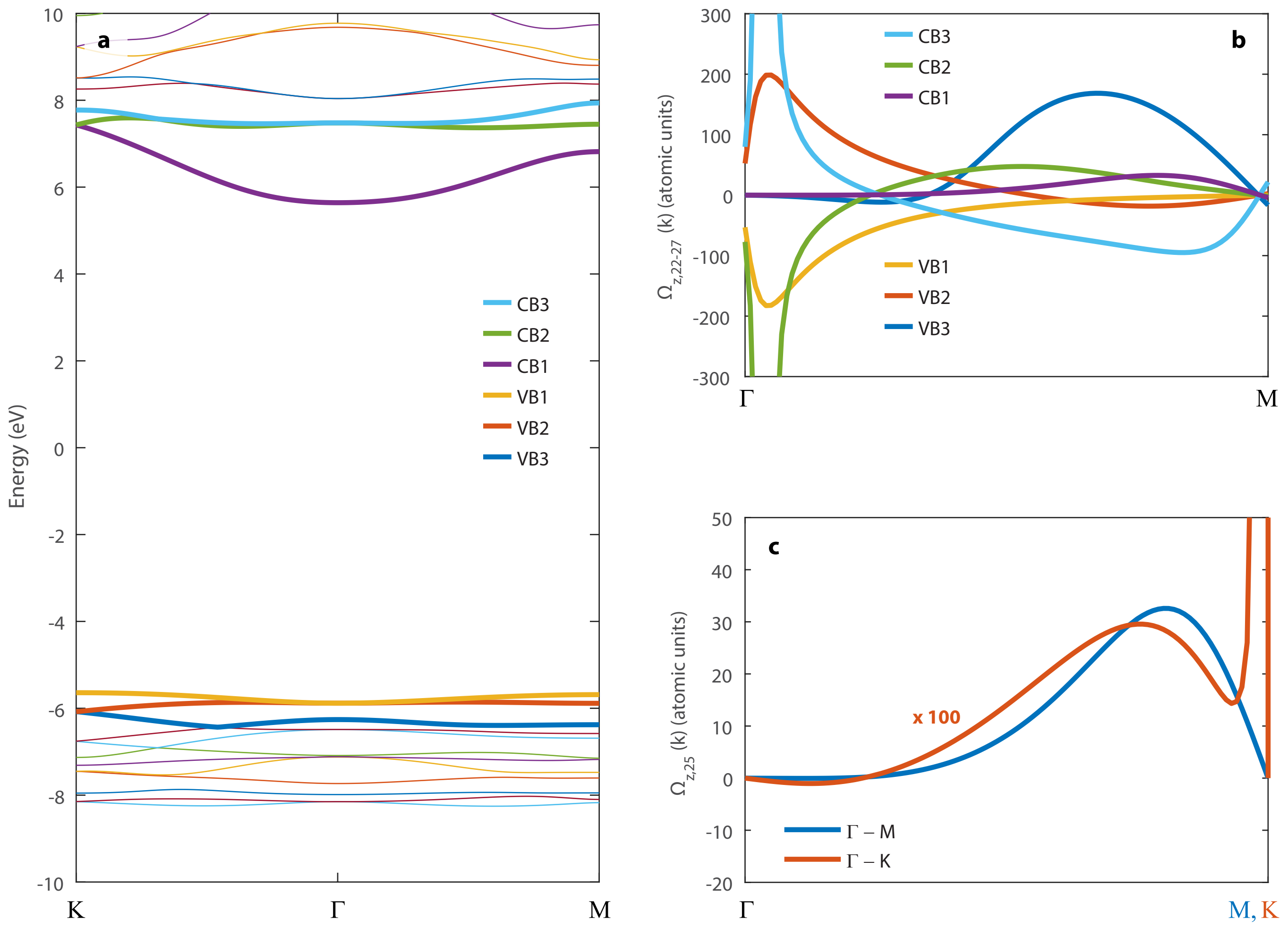

Supplementary Figure 6. **Bandstructure and Berry curvature for different bands/directions.** **a**, Zoomed-in bandstructure calculated using MGGA-TB09LDA. A few representative bands are highlighted. **b**, Berry curvature of the highlighted bands in **a**. **c**, Comparison of Berry curvature of the first conduction band in $\Gamma - \mathrm{M}$ and $\Gamma - \mathrm{K}$ direction. Note that the value in $\Gamma - \mathrm{K}$ direction (should be zero due to symmetry reason) has been multiplied by 100 to be visible in the same plot.

Intensity calibration

- Multi-channel plate and phosphor's screen are assumed to have a flat spectral response in our measurement range.
- Polarizer reflectivity: for S-pol and P-pol are calculated using [14] as discussed before.
- Grating reflectivity: the grating efficiency for the two different polarizations (S-pol and P-pol) are obtained from the manufacturer and applied to the corresponding cases.
- Effect of slit on the total spectral intensity: since the different wavelengths diffract differently, the spatio-spectral distribution of the beam after the slit could be illustrated in Supplementary Figure 8. Therefore, the beams carrying high-energy photons have a smaller divergence, thus the percentage of transmission is higher than for low-energy photons. As a first order calibration to this effect, we use the following formula:

$$\mathrm{S}(\omega)_{\text{averaged after slit}} = \mathrm{S}(\omega)_{\text{averaged before slit}} \cdot \omega^2 \tag{33}$$

- First order propagation approximation and beam profile averaging effect: because we measured only the spectra, the phase was not measured, thus we could not perform the back propagation to get the exact spatially resolved EUV spectra at the focus. Therefore, we assume the averaged spectral intensity measured before the slit is the

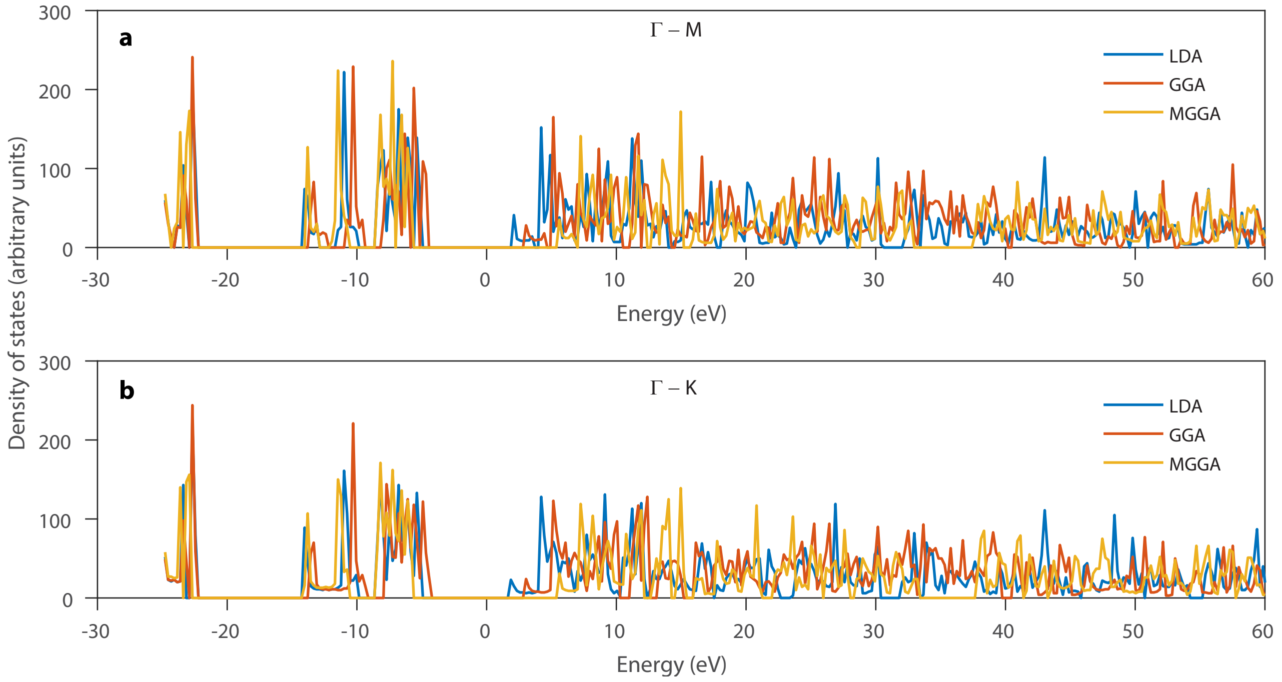

Supplementary Figure 7. **Density of states of α–quartz for different directions and approximations. a, b,** Density of states calculated from the bandstructure for two directions $\Gamma - \mathrm{M}$ and $\Gamma - \mathrm{K}$ respectively, and for different approximations.

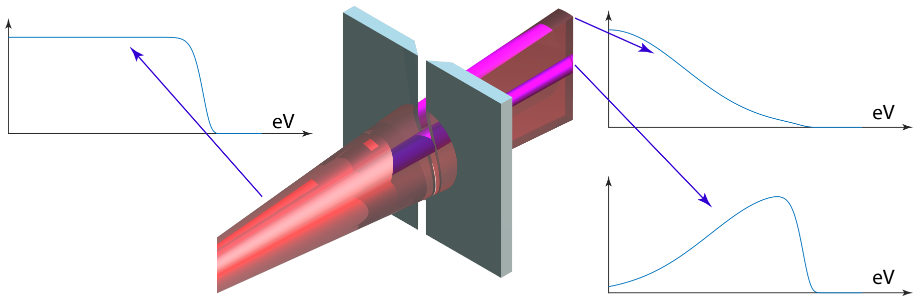

Supplementary Figure 8. **Spatio-spectral distribution before and after the slit**. The spatially-integrated model spectrum before the slit is shown on the left. Spectra at the inner and outer part of the transmitted beam are shown on the right.

signal at the center of the beam (the degree of nonlinearity is high). First order propagation inside the crystal is approximated by: $S(\omega)_{\text{propagated}} = S(\omega)_{\text{microscopic}} \cdot \omega^2$. As a consequence, we have:

$$\mathrm{S}(\omega)_{\text{measured on MCP}} = \mathrm{S}(\omega)_{\text{microscopic}} \cdot \omega^4 \cdot \mathrm{R}(\omega)_{\text{grating}} \tag{34}$$

- Electric field strength: is calculated using the measurement of the beam profile at the focus for various intensity settings and the temporal characterization of the electric field using Transient Grating - Frequency-Resolved Optical Gating.

Fitting of the Berry curvature

Basis of the fitting: Associated spectra of different Fourier coefficients of the Berry curvature

In order to examine the reliability of the fitting, we make use of the analytical derivation of the associated spectra of the Berry curvature: Supplementary Eq. 25, 26. Although the derivations apply for the long pulse regime, because we are only interested in the relative intensity of the harmonic peaks, not their shapes, these equations

are of great applicability. Indeed, we could assign Supplementary Eq. 25 as $v_\nu(t)_\perp = \frac{e\mathrm{E}_0}{\hbar}\sum_{n=1}^{\infty}\gamma_n \mathrm{A}_n$ with $\mathrm{A}_n = -\sum_{m=1}^{\infty}(-1)^m J_{2m-1}(na\frac{e}{\hbar}\frac{\mathrm{E}_0}{\omega_\mathrm{L}})\big(\sin[2m\omega_\mathrm{L}t] + \sin[(2m-2)\omega_\mathrm{L}t]\big)$. Now we could plot associated spectra of different $\{\mathrm{A}_n\}$ as illustrated in Supplementary Figure 9 for the electric field strength of $\mathrm{E}_0 = 1.0$ V/Å. Due to the properties of the Bessel functions, the summation over m converges at $m = 30$ for our energy range.

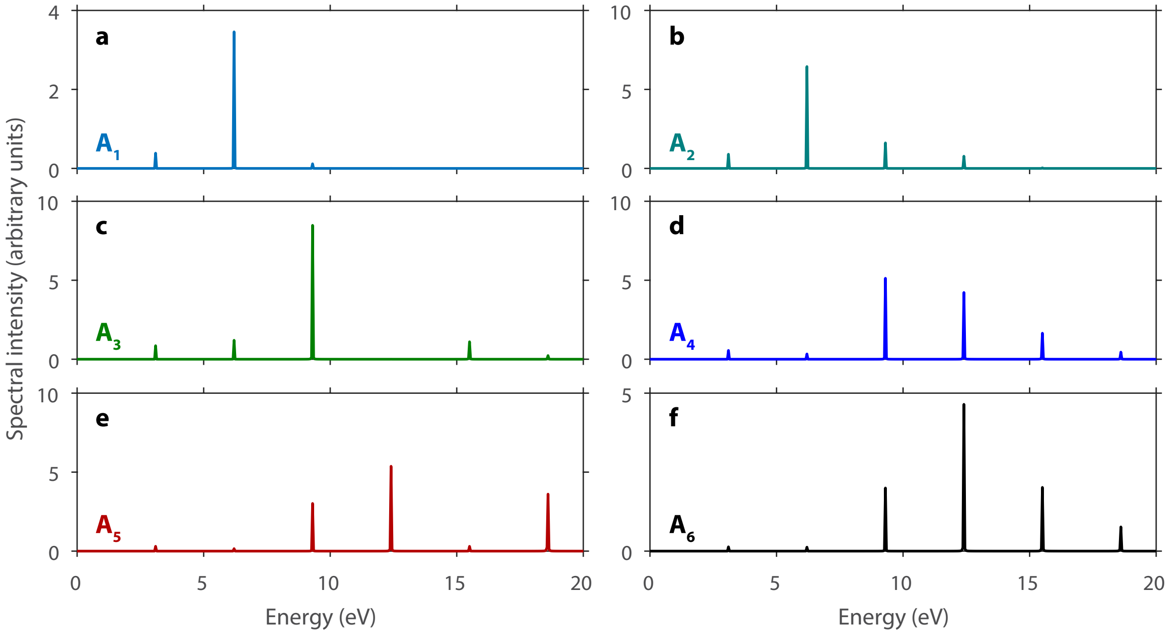

Supplementary Figure 9. **Fourier coefficients of Berry curvature form a basis set of spectra**. **a** → **f**, Associated spectra of the individual components of $\{\mathrm{A}_n\}$ for $n = 1 \to 6$.

From Supplementary Figure 9, it is clear that: (i) all A_n exhibit only even harmonics, with different number of harmonics as well as their relative intensity; (ii) the higher the number n is, the higher the associated photon energy is. The first feature is obvious from the formula while the second feature is a consequence of the Bessel functions. Therefore, $\{\mathrm{A}_n\}$ forms a basis set of HHG spectra from Berry curvature. This basis set is possibly complete but redundant because the harmonics of A_n at high n are very similar. In addition, within our photon energy range, all A_n with $n > 20$ contribute very little and thus play almost no role in the fitting result.

In conclusion, the above analysis shows that the basis set formed by the series $\{\mathrm{A}_n\}$ could be well utilized for retrieving the Berry curvature from our measured HHG spectra, especially for the range of $n = 1 \to 10$.

The fitting

We first simulated the $\mathbf{k}(t)$ using the measured electric field and a given set of dipole matrix elements. The fitting is done using Eq. 2 (main text) and the trust-region reflective algorithm in a commercial software package. The fitting error is defined as:

$$G = \sum_{\omega}\left[S(\omega)_{\text{microscopic}} - S(\omega)_{\text{Berry curvature}}\right]^2 \tag{35}$$

Since the basis set used above is not orthonormal, there are different sets of Fourier coefficients that give similar fitting results. Therefore, in order to quantify more accurately the retrieved Berry curvature, we performed a large number (10^4) of fittings using random-number generation for the initial guesses. The fitted results with errors lower than a given threshold are then used to illustrate the probability density of the retrieved Berry curvature given in Fig. 3b main text. More accurate measurements and calibration of this experiment could be carried out in the future, for instance with atomically-thin quartz samples, which could help deriving a more accurate effective Berry curvature in quartz. Unfortunately such samples are not presently available.

*trung.luu@phys.chem.ethz.ch

SUPPLEMENTARY REFERENCES

[1] Robert W. Boyd, *Book* (Academic Press, 2008) p. 640.
[2] D. Golde, T. Meier, and S. W. Koch, "High harmonics generated in semiconductor nanostructures by the coupled dynamics of optical inter- and intraband excitations," Physical Review B - Condensed Matter and Materials Physics **77**, 1–6 (2008).
[3] O. Schubert, M. Hohenleutner, F. Langer, B. Urbanek, C. Lange, U. Huttner, D. Golde, T. Meier, M. Kira, S. W. Koch, and R. Huber, "Sub-cycle control of terahertz high-harmonic generation by dynamical Bloch oscillations," Nature Photonics **8**, 119–123 (2014).
[4] Shambhu Ghimire, Anthony D. DiChiara, Emily Sistrunk, Pierre Agostini, Louis F. DiMauro, and David A. Reis, "Observation of high-order harmonic generation in a bulk crystal," Nature Physics **7**, 138–141 (2011).
[5] Shambhu Ghimire, Anthony D. Dichiara, Emily Sistrunk, Georges Ndabashimiye, Urszula B. Szafruga, Anis Mohammad, Pierre Agostini, Louis F. Dimauro, and David A. Reis, "Generation and propagation of high-order harmonics in crystals," Physical Review A - Atomic, Molecular, and Optical Physics **85**, 1–6 (2012).
[6] M. Wegener, *Extreme Nonlinear Optics*, Advanced Texts in Physics (Springer-Verlag, Berlin/Heidelberg, 2005).
[7] Milton Abramowitz and Irene A Stegun, *National Bureau of Standards - Applied Mathematics Series* (1964) p. 958.
[8] T T Luu, M Garg, S Yu Kruchinin, A Moulet, M Th Hassan, and E Goulielmakis, "Extreme ultraviolet high-harmonic spectroscopy of solids," Nature **521**, 498–502 (2015).
[9] Mermin and Ashcroft, *Physics* (Brooks/Cole Cengage Learning, 1976) p. 826.
[10] Felix Bloch, "Ueber die Quantenmechanik der Elektronen in Kristallgittern," Zeitschrift fuer Physik **52**, 555–600 (1929).
[11] M S Dresselhaus, G Dresselhaus, and A Jorio, *Group Theory* (Springer Berlin Heidelberg, Berlin, Heidelberg, 2008).
[12] Hanzhe Liu, Yilei Li, Yong Sing You, Shambhu Ghimire, Tony F Heinz, and David A Reis, "High-harmonic generation from an atomically thin semiconductor," Nature Physics **13**, 262–265 (2016).
[13] Oliver D. Mücke, "Isolated high-order harmonics pulse from two-color-driven Bloch oscillations in bulk semiconductors," Physical Review B **84**, 081202 (2011).
[14] M Sanchez Rio and R J Dejus, "XOP : A Multiplatform Graphical User Interface for Synchrotron Radiation Spectral and Optics Calculations ." Spie **3152**, 148–157 (1997).
[15] M Gradhand, D V Fedorov, F Pientka, P Zahn, I Mertig, and B L Györffy, "First-principle calculations of the Berry curvature of Bloch states for charge and spin transport of electrons," Journal of Physics: Condensed Matter **24**, 213202 (2012).
[16] M. V. Berry, "Quantal Phase Factors Accompanying Adiabatic Changes," Proceedings of the Royal Society A: Mathematical, Physical and Engineering Sciences **392**, 45–57 (1984).
[17] Di Xiao, Ming Che Chang, and Qian Niu, "Berry phase effects on electronic properties," Reviews of Modern Physics **82**, 1959–2007 (2010).
[18] G Y Guo, Yugui Yao, and Qian Niu, "Ab initio Calculation of the Intrinsic Spin Hall Effect in Semiconductors," Physical Review Letters **94**, 226601 (2005).
[19] Y Yao and Z Fang, "Sign Changes of Intrinsic Spin Hall Effect in Semiconductors and Simple Metals: First-Principles Calculations," Physical Review Letters **95**, 156601 (2005).
[20] Takahiro Fukui, Yasuhiro Hatsugai, and Hiroshi Suzuki, "Chern Numbers in Discretized Brillouin Zone: Efficient Method of Computing (Spin) Hall Conductances," Journal of the Physical Society of Japan **74**, 1674–1677 (2005).
[21] Takahiro Fukui and Yasuhiro Hatsugai, "Quantum Spin Hall Effect in Three Dimensional Materials: Lattice Computation of Z 2 Topological Invariants and Its Application to Bi and Sb," Journal of the Physical Society of Japan **76**, 053702 (2007).
[22] P D Sacramento, M A N Araújo, and E V Castro, "Hall conductivity as bulk signature of topological transitions in superconductors," EPL (Europhysics Letters) **105**, 37011 (2014), arXiv:arXiv:1302.3122v1.
[23] Jürgen Kübler and Claudia Felser, "Berry curvature and the anomalous Hall effect in Heusler compounds," Physical Review B **85**, 012405 (2012).
[24] Mads Brandbyge, José-Luis Mozos, Pablo Ordejón, Jeremy Taylor, and Kurt Stokbro, "Density-functional method for nonequilibrium electron transport," Physical Review B **65**, 165401 (2002).
[25] José M Soler, Emilio Artacho, Julian D Gale, Alberto García, Javier Junquera, Pablo Ordejón, and Daniel Sánchez-Portal, "The SIESTA method for ab initio order- N materials simulation," Journal of Physics: Condensed Matter **14**, 2745–2779 (2002).
[26] Fabien Tran and Peter Blaha, "Accurate Band Gaps of Semiconductors and Insulators with a Semilocal Exchange-Correlation Potential," Physical Review Letters **102**, 226401 (2009).
[27] T.H. DiStefano and D.E. Eastman, "The band edge of amorphous SiO2 by photoinjection and photoconductivity measurements," Solid State Communications **9**, 2259–2261 (1971).
[28] Z. A. Weinberg, G. W. Rubloff, and E. Bassous, "Transmission, photoconductivity, and the experimental band gap of thermally grown SiO2 films," Physical Review B **19**, 3107–3117 (1979).
[29] Peter Blaha, Karlheinz Schwarz, Georg K H Madsen, Dieter Kvasnicka, and Joachim Luitz, "WIEN2k - An Augmented PlaneWave + Local Orbitals Program for Calculating Crystal Properties," (2010).

[30] Georges Ndabashimiye, Shambhu Ghimire, Mengxi Wu, Dana A. Browne, Kenneth J. Schafer, Mette B. Gaarde, and David A. Reis, "Solid-state harmonics beyond the atomic limit," Nature **534**, 520–523 (2016).

[31] Mengxi Wu, Shambhu Ghimire, David A. Reis, Kenneth J. Schafer, and Mette B. Gaarde, "High-harmonic generation from Bloch electrons in solids," Physical Review A **91**, 043839 (2015).
